# Association of High-Sensitivity C-Reactive Protein (hs-CRP) with Weight Loss After Sleeve Gastrectomy and Roux-en-Y Gastric Bypass at 10 Years: A Secondary Analysis of the SLEEVEPASS Randomized Clinical Trial

**DOI:** 10.1007/s11695-024-07567-w

**Published:** 2024-11-07

**Authors:** Ilmari Saarinen, Marjatta Strandberg, Saija Hurme, Sofia Grönroos, Anne Juuti, Mika Helmiö, Paulina Salminen

**Affiliations:** 1 University of Turku, Turku, Finland; 2 Satasairaala Central Hospital, Pori, Finland; 3 Turku University Hospital, Turku, Finland; 4 Helsinki University Hospital, Helsinki, Finland; 5 University of Helsinki, Helsinki, Finland

**Keywords:** Metabolic bariatric surgery, Sleeve gastrectomy, Roux-en-Y gastric bypass, Severe obesity, Chronic inflammation, High-sensitivity CRP, Hs-CRP

## Abstract

**Background:**

Severe obesity is associated with a low-grade chronic inflammation, and high-sensitivity C-reactive protein (hs-CRP) is a marker that can be used to evaluate chronic inflammation status. Metabolic bariatric surgery (MBS) is shown to decrease hs-CRP level, but long-term results are scarce, and association with weight loss outcomes is undetermined. This study aims to evaluate chronic inflammation in patients with obesity using hs-CRP, and its association with long-term weight loss outcomes after laparoscopic sleeve gastrectomy (LSG) and laparoscopic Roux-en-Y gastric bypass (LRYGB).

**Methods:**

The long-term follow-up data of SLEEVEPASS (ClinicalTrials.gov NCT00793143) randomized clinical trial (RCT) was used. Hs-CRP was measured at baseline, and at 6 months, 1, 3, 5, 7, and 10 years after surgery, and the association with weight and weight loss outcomes were analyzed.

**Results:**

Hs-CRP at baseline was available for 59 out of 240 (24.6%) patients. In the whole study population, the nadir hs-CRP (mean estimate 1.14 mg/ml, 95% CI 0.87–1.49) was achieved at 3 years after surgery with a statistically significant difference to baseline (*p* = 0.003). No statistically significant difference was seen between LSG and LRYGB in hs-CRP change over time (operation*time interaction *p* = 0.540). Higher hs-CRP correlated with higher BMI at baseline (Spearman correlation 0.282, *p* = 0.030) and at 10 years (Spearman correlation 0.490, *p* = 0.001). At 10 years, a greater percentage total weight loss (%TWL) correlated with lower hs-CRP level (Spearman correlation − 0.558, *p* < 0.001). Baseline hs-CRP (Spearman correlation − 0.152, *p* = 0.299) and hs-CRP change in first 6 months postoperatively (Spearman correlation 0.167, *p* = 0.254) did not correlate statistically significantly with %TWL at 10 years.

**Conclusions:**

MBS decreases hs-CRP also at long-term follow-up with weight loss as the driving force. Neither baseline hs-CRP nor hs-CRP change at 6 months were feasible as a predictive marker for long-term outcomes.

## Introduction

Obesity and its comorbidities such as type 2 diabetes (T2DM) and cardiovascular diseases (CVDs) are among the biggest global health threats [1]. Low-grade chronic inflammation is shown to be associated with obesity [2–5]. It has been suggested that adipose tissue activates and produces proinflammatory cytokines such as tumor necrosis factor alpha (TNF-α) and interleukin 6 (IL-6) [6, 7], and IL-6 in turn causes increased production of C-reactive protein (CRP), a commonly used inflammatory marker [8]. High-sensitivity CRP (hs-CRP) has been used to assess low-grade inflammatory state in patients with metabolic diseases. Elevated hs-CRP levels have been reported in patients with obesity [9, 10]. Hs-CRP has predictive value in CVDs [11, 12], and therefore The American Heart Association recommends it for clinical use as one parameter of CVD risk [13]. Many studies have shown that elevated hs-CRP may also be an independent risk factor for worsening insulin resistance, increased mortality of patients with T2DM, and de novo T2DM [14–17]. However, there are also studies suggesting that obesity may be the link between elevated hs-CRP and metabolic diseases [18].

Metabolic bariatric surgery (MBS) is the most efficient treatment of severe obesity with good and sustainable weight loss and beneficial effects on obesity-related comorbidities [19–23]. MBS has also been shown to decrease hs-CRP levels [24]. A recent study suggests that hs-CRP correlates with preoperative weight and postoperative weight loss but not with remission of metabolic diseases [25]. Since 2014, laparoscopic sleeve gastrectomy (LSG) has been the most common bariatric procedure globally surpassing the gold standard, laparoscopic Roux-en-Y gastric bypass (LRYGB) in the number of performed operations [26]. Most of the studies examining MBS and hs-CRP focus on short-term results of LRYGB, and long-term results on the effects of LSG and both procedures on hs-CRP are lacking. The potential of hs-CRP as a predictive marker of MBS outcomes is yet to be determined [25].

To our knowledge, the SLEEVEPASS randomized clinical trial (RCT) is so far the largest RCT comparing the results of LSG and LRYGB with a 10-year follow-up [27]. The aims of this 10-year secondary analysis of the SLEEVEPASS trial were to assess the effect of MBS on hs-CRP at long-term follow-up and the association between weight loss and hs-CRP, to evaluate the potential predictive value of hs-CRP for MBS outcomes, and to compare the effects of LSG and LRYGB on hs-CRP.

## Materials and Methods

The study design, rationale, and methods of the SLEEVEPASS trial have been reported previously [27]. Briefly, the SLEEVEPASS trial is a multicenter, multisurgeon, open label, randomized clinical equivalence trial conducted between March 2008, and June 2010 in three hospitals in Finland (Turku, Vaasa, and Helsinki), randomizing 240 patients with severe obesity to undergo either LSG or LRYGB. Eligibility criteria included body mass index (BMI; kg/m^2^) > 40 or > 35 with an obesity-associated comorbidity, age 18 to 60 years, and a previous failed adequate conservative treatment. Exclusion criteria were BMI greater than 60, significant eating or psychiatric disorder, active alcohol or substance abuse, active gastric ulcer disease, severe gastroesophageal reflux disease (GERD) with a large hiatal hernia, and previous bariatric surgery. The 10-year follow-up of the trial was completed in January 2021. The study protocol was approved by the ethics committees of each participating hospital (Turku, Helsinki, and Vaasa). The trial was designed in accordance with the principles of the Declaration of Helsinki and registered at the clinical trials registry of the National Institutes of Health (ClinicalTrials.gov NCT00793143). All patients gave written informed consent.

CRP values were assayed with high-sensitivity CRP test (S-uCRP or S-hs-CRP) from blood samples collected from the trial patients preoperatively and at 6 months, 1, 3, 5, 7, and 10 years. All available hs-CRP values at all time points were included with the vast majority of the available values were from the patient cohort at Turku University hospital and a minority from Helsinki University Hospital. Hs-CRP tests were not included in the laboratory tests taken at Vaasa Central Hospital. All hs-CRP values ≥ 25 were considered as signs of an acute infection or autoimmune disease activation and were therefore excluded from the analyses. Weight loss was defined as percentage total weight loss (%TWL [preoperative weight − postoperative weight/preoperative weight × 100]).

### Statistical Analyses

Continuous variables were characterized using means and standard deviations (SD) or medians with 25th and 75th percentiles (Q1, Q3) in case of non-normally distributed variables. For categorical variables, frequencies and percents were used.

Linear mixed models suitable for repeated measures were used to evaluate the differences between time-points and operations in hs-CRP. The final model included operation, time, BMI, and interaction of operation and time. The interaction of operation and time was not statistically significant and therefore the results are presented using main effects of operation and time. The results were quantified using model-based estimates with 95% confidence intervals (95% CI). Values of hs-CRP were not normally distributed, and logarithmic transformation was used in the analyses, and estimates were transformed back to the original scale. Tukey–Kramer method was used to correct *p* values of pairwise comparisons of timepoints.

The correlations between continuous variables were evaluated using Spearman’s rank-order correlation coefficient. Two-tailed *p* values less than 0.05 were considered statistically significant. Statistical analyses were performed using SAS software, version 9.4 for Windows (SAS Institute Inc., Cary, NC, USA).

## Results

**Fig. 1:**
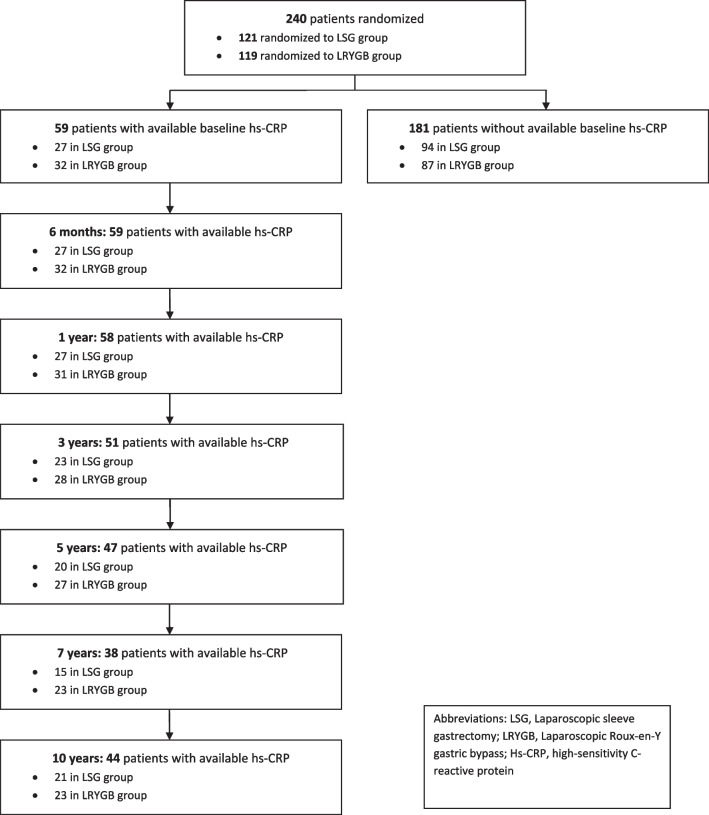
Flow of patients. Abbreviations: LSG, laparoscopic sleeve gastrectomy; LRYGB, laparoscopic Roux-en-Y gastric bypass; Hs-CRP, high-sensitivity C-reactive protein

The flow of the patients is presented in Fig. 1. Of the 240 SLEEVEPASS study patients, 59 (24.6%) had baseline hs-CRP value available (56 patients at Turku University Hospital, 3 patients at Helsinki University hospital). Out of these 59 patients, 27 underwent LSG and 32 LRYGB. Baseline characteristics of the patients in this secondary analysis are described in Table 1.

**Table 1:** Baseline patient characteristics

	LSG	LRYGB	All included patients	Excluded patients	*P* comparing included and excluded patients
*N*	27	32	59	181	NA
Age (years), mean (SD)	46.7 (8.6)	47.2 (10.1)	47.0 (9.4)	48.9 (9.3)	0.945†
Female, frequency (%)	18/27 (66.7%)	20/32 (62.5%)	38/59 (64.4%)	129/181 (71.3%)	0.332*
Male, frequency (%)	9/27 (33.3%)	12/32 (37.5%)	21/59 (35.6%)	52/181 (28.7%)	0.332*
Body mass index, BMI (kg/m^2^), mean (SD)	47.3 (5.9)	48.9 (7.8)	48.2 (7.0)	47.8 (6.9)	0.896†
T2D, frequency (%)	13/27 (48.2%)	15/32 (46.9%)	28/59 (47.5%)	73/181 (40.3%)	0.364*
Blood pressure medication, frequency (%)	15/27 (55.6%)	24/32 (75.0%)	39/59 (66.1%)	131/181 (72.4%)	0.410*
High LDL/cholesterol medication, frequency (%)	7/27 (25.9%)	11/32 (34.4%)	18/59 (30.5%)	66/181 (36.5%)	0.436*
CPAP-treated OSA, frequency (%)	5/27 (18.5%)	10/32 (31.3%)	15/59 (25.4%)	48/181 (26.5%)	0.134*
Hs-CRP (mg/L), median (Q_1_-Q_3_)	5.5 (2.7–8.2)	4.0 (2.8–5.5)	4.2 (2.8–7.0)	NA	NA

*LSG l*aparoscopic sleeve gastrectomy, *LRYGB* laparoscopic Roux-en-Y gastric bypass, *T2D* type 2 diabetes mellitus, *LDL* low-density lipoprotein, C*PAP* continuous positive airway pressure, *OSA* obstructive sleep apnea, *Hs-CRP* high-sensitivity C-reactive protein

*Fisher’s exact test

†Analysis of variance

**Fig. 2:**
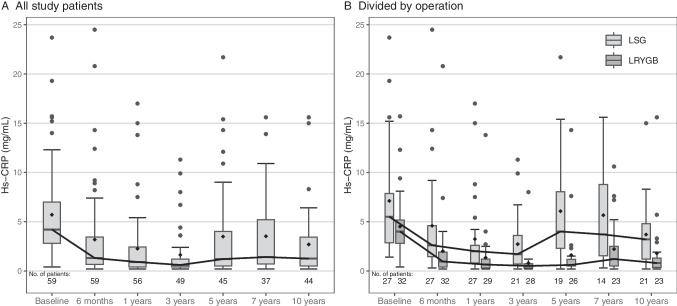
Hs-CRP through a 10-year follow-up. **A** All study patients. **B** Divided by operation. The lower and upper hinges correspond to the first and third quartiles (the 25th and 75th percentiles), and whiskers indicate smallest observation greater than or equal to lower hinge—1.5 * IQR and largest observation less than or equal to upper hinge + 1.5 * IQR. Dots indicate outliers. Abbreviations: LSG, laparoscopic sleeve gastrectomy; LRYGB, laparoscopic Roux-en-Y gastric bypass; Hs-CRP, high-sensitivity C-reactive protein

Hs-CRP at all time points in the whole study population is presented in Fig. 2A, and hs-CRP levels by operation are presented in Fig. 2B. Hs-CRP was the highest at baseline (mean estimate 2.06, 95% CI 1.48–2.86). Postoperatively, a decrease was seen, and the nadir hs-CRP was achieved at 3 years (mean estimate 1.14 mg/L, 95% CI 0.87–1.49). There were statistically significant differences in hs-CRP between timepoints (*p* < 0.001). Compared to baseline, there was a statistically significant difference in hs-CRP only at 3 years (*p* = 0.043). After 3 years, hs-CRP increased until 7 years (mean estimate 1.90 mg/L, 95% CI 1.42–2.56), but decreased again at 10 years (mean estimate 1.38 mg/L, 95% CI 1.05–1.81). There was no statistically significant difference between the operations in hs-CRP change over time (operation*time interaction *p* = 0.540). Hs-CRP decreased after both procedures, but it was higher through the whole 10-year follow-up in LSG group (main effect of operation *p* < 0.001). Results were adjusted with BMI to enable the evaluation of the effect of operation. The results of the final model of Hs-CRP through 10-year follow-up are presented in Table 2.

**Table 2:** Mean hs-CRP at follow-up points and effects of operation and time

All study patients					
**Follow-up point**	Hs-CRP (mg/mL), mean estimate (95% CI)*	***P* compared to baseline†**		***P* compared to 3 years†**	
**Baseline**	2.06 (1.48–2.86)	NA		0.043	
**6 months**	1.92 (1.49–2.48)	0.999		0.002	
**1 year**	1.42 (1.09–1.85)	0.518		0.680	
**3 years**	1.14 (0.87–1.49)	0.043		NA	
**5 years**	1.81 (1.38–2.38)	0.994		0.026	
**7 years**	1.90 (1.42–2.56)	0.999		0.023	
**10 years**	1.38 (1.05–1.81)	0.286		0.858	
**Operation comparison**					
		Main effect of operation*	Main effect of time*		Operation*time interaction*
**LSG**	2.5 (1.48–3.4)	< 0.001	0.001		0.540
**LRYGB**	1.0 (0.8–1.4)				

*LSG* laparoscopic sleeve gastrectomy, *LRYGB* laparoscopic Roux-en-Y gastric bypass, *Hs*-*CRP* high-sensitivity C-reactive protein

*Linear mixed models were used. Results were adjusted for BMI. Logarithmic transformation was used in the analyses, and estimates were transformed back to the original scale

†Tukey–Kramer method

A greater 10 year-%TWL correlated statistically significantly with lower hs-CRP at 10 years (Spearman correlation − 0.558, *p* < 0.001). The hs-CRP at baseline correlated statistically significantly with preoperative BMI (Spearman correlation 0.282, *p* = 0.030). Also at 10 years, hs-CRP correlated statistically significantly with present BMI (Spearman correlation 0.490, *p* = 0.001). No statistically significant correlation was seen between baseline hs-CRP level and %TWL at 10 years (Spearman correlation − 0.152, *p* = 0.299). Similarly, hs-CRP change in first 6 months postoperatively (Spearman correlation 0.167, *p* = 0.254) did not correlate statistically significantly with %TWL at 10 years.

## Discussion

In this secondary analysis of the SLEEVEPASS trial, metabolic bariatric surgery resulted in sustainable improvement on chronic inflammation evaluated by decreased postoperative hs-CRP levels up to 10 years postoperatively with both the nadir and statistically significant difference at 3 years. Between LSG and LRYGB, there was no statistically significant difference in hs-CRP change over time. Hs-CRP correlated statistically significantly with BMI at baseline and at 10 years. In addition, greater %TWL correlated statistically significantly with lower hs-CRP at 10 years. Preoperative hs-CRP and hs-CRP change in first 6 months postoperatively did not correlate statistically significantly with 10-year %TWL outcomes.

To our knowledge, this is the first RCT comparing LSG and LRYGB to assess post-bariatric hs-CRP decrease with a 10-year follow-up. Other studies have also reported sustainable improvement in hs-CRP level at 7 years with a slight increase from the lowest point at 3 years to 7 years [28, 29]. This is in line with this study having the nadir hs-CRP at 3 years and other studies reporting lowest hs-CRP levels at 2 years postoperatively followed by a minor increase [28, 29]. Our results indicate and corroborate a strong correlation between hs-CRP and BMI pre- and postoperatively. Weight loss seems to be the driving force behind the hs-CRP decrease after MBS [25, 28, 30]. These findings support the hypothesis that the amount of adipose tissue in obesity is the key determinant of the grade of chronic inflammation [4, 18].

In this study, baseline hs-CRP was not a predictive factor of %TWL at 10 years. In a previous study [25], higher baseline hs-CRP was associated with greater weight loss at 3 years postoperatively. However, this correlation was low and not considered to have clinical utility [25]. We also examined whether hs-CRP change during the first 6 months after surgery had predictive value over long-term outcome of surgery as the majority of the MBS-induced weight loss is achieved during this time, but the change in hs-CRP during the first 6 months did not correlate statistically significantly with long-term outcomes. The applicability of baseline hs-CRP as a prognostic tool after MBS is very limited based on the results of this study, but this serum biomarker should be included in future trials to further assess the clinical applicability.

In this RCT, baseline hs-CRP was higher in LSG group, and this difference persisted through the follow-up to 10 years. When adjusted for BMI, operation*time interaction was not statistically significant. Our results are in line with a retrospective study [28] showing no differences in hs-CRP between the two procedures. In a recent study comparing serum biomarkers between laparoscopic adjustable banding and LRYGB [25], the hs-CRP decrease independent of weight loss was greater after LRYGB, highlighting the potential impact of performed anatomical modifications.

Our study has several limitations. Firstly, our major limitation is the small number of patients with available hs-CRP values at baseline and at all follow-up time points. This is mainly due to the fact of missing laboratory tests due to human error at two of the participating study hospitals as Vaasa has no hs-CRP values, and in Helsinki, only three patients have available hs-CRP values available. However, as this is based on the simple error of not including the hs-CRP in the follow-up, selection bias is very unlikely. All of the results may be naturally influenced by the small number of patients. However, out of the 80 patients at Turku University Hospital, 53 (66%) patients had available hs-CRP values. Secondly, our small sample size did not allow assessment of potential association between hs-CRP and secondary endpoints of MBS, such as resolution of comorbidities and mortality. Thirdly, the higher baseline hs-CRP in the LSG group may be and most likely is caused by the small sample size in this randomized data. Fourthly, only the 3-year hs-CRP differed statistically significantly from baseline and other follow-up points, and this may also be attributable to the sample size. However, our results form a similar hs-CRP trajectory compared with other studies [28, 29]. The fifth limitation is that this study consists of patients with Caucasian ethnic background potentially limiting the generalizability of the results. Further research in larger prospective cohorts is needed to evaluate the potential association between hs-CRP and MBS outcomes.

In summary, our findings indicate that MBS decreases hs-CRP also at long-term follow-up with weight loss as the driving force demonstrating a long-term enhancement in chronic inflammation after both LSG and LRYGB. Neither hs-CRP at baseline nor hs-CRP change during the first 6 months after surgery was feasible as a predictive marker for long-term outcomes of MBS.

## Data Availability

The data will be made available by reasonable request from the corresponding author.
